# PEER TO PEER SUPPORT AND HEALTH CARE UTILIZATION AMONG COMMUNITY-DWELLING OLDER ADULTS

**DOI:** 10.1093/geroni/igz038.3151

**Published:** 2019-11-08

**Authors:** Elizabeth A Jacobs, Rebecca Schwei, Scott Hetzel, Jane Mahoney, KyungMann Kim

**Affiliations:** 1 The University of Texas at Austin Dell Medical School, Austin, Texas, United States; 2 University of Wisconsin-Madison School of Medicine and Public Health, Madison, Wisconsin, United States; 3 University of Wisconsin-Madison Biostatistics & Medical Informatics, Madison, Wisconsin, United States; 4 University of Wisconsin School of Medicine and Public Health, Madison, Wisconsin, United States

## Abstract

The majority of older adults want to live and age in their communities. Some community-based organizations (CBOs) have initiated peer-to-peer support services to promote aging in place but the effectiveness of these programs is not clear. Our objective was to compare the effectiveness of a community-designed and implemented peer-to-peer support program vs. access to standard community services, in promoting health and wellness in vulnerable older adult populations. We partnered with three CBOs, one each in California, Florida, and New York, to enroll adults 65 > years of age who received peer support and matched control participants (on age, gender, and race/ethnicity) in an observational study. We followed participants over 12 months, collecting data on self-reported urgent care and emergency department visits and hospitalizations. In order to account for the lack of randomization, we used a propensity score method to compare outcomes between the two groups. We enrolled 222 older adults in the peer-to-peer group and 234 in the control group. After adjustment, we found no differences between the groups in the incidence of hospitalization, urgent and emergency department visits, and composite outcome of any health care utilization. The incidence of urgent care visits was statistically significantly greater in the standard community service group than in the peer-to-peer group. Given that the majority of older adults and their families want them to age in place, the question of how to do this is highly relevant. Peer-to-peer services may provide some benefit to older adults in regard to their health care utilization.

